# Ticking off Lyme disease: OspA mRNA vaccine halts infection in mouse model

**DOI:** 10.1016/j.omtn.2025.102564

**Published:** 2025-07-31

**Authors:** Wei He, Amy Rasley, Nicholas O. Fischer, Jessica Z. Kubicek-Sutherland, Zachary R. Stromberg

**Affiliations:** 1 Physical and Life Sciences Directorate, Lawrence Livermore National Laboratory, Livermore, CA, USA; 2 Physical Chemistry and Applied Spectroscopy, Los Alamos National Laboratory, Los Alamos, NM, USA; 3 Chemical and Biological Signatures Group, Pacific Northwest National Laboratory, Richland, WA, USA

## Main text

Lyme disease, a condition caused by *Borrelia burgdorferi* sensu lato and transmitted to humans via ticks, affects approximately 676,000 individuals annually in the United States and Western Europe.^1^ Currently, there is no approved Lyme vaccine available for human use. A promising study by Tahir et al., published in *Molecular Therapy Nucleic Acids*, investigated the efficacy of mRNA and subunit vaccines targeting Lyme disease.^2^ This research demonstrated complete protection from infection in a tick-fed mouse model using an outer surface protein A (OspA) mRNA vaccine. Although mRNA vaccines have shown success against viral pathogens, their clinical application against bacterial diseases has been limited.^3^ Thus, this study represents an important step toward developing an effective mRNA vaccine against Lyme disease.

The OspA protein in *B*. *burgdorferi* aids in tick colonization by facilitating adherence to tick midgut cells. Vaccines such as LYMErix (previously withdrawn from human use) and Recombitek Lyme for canines have utilized OspA to elicit immune responses to block transmission. In addition, companies are progressing vaccines through clinical trials, such as an mRNA candidate in a phase 1/2 randomized, observer-blind, placebo-controlled clinical trial by Moderna (NCT05975099) and the multivalent recombinant protein vaccine VLA15 in a randomized, observer-blind, placebo-controlled phase 3 study (Vaccine Against Lyme for Outdoor Recreationists) by Valneva and Pfizer (NCT05477524). Challenges arise in expressing bacterial antigens in mammalian systems, including deciding the optimal location for antigen expression—whether cytoplasmic, membrane-bound, or secreted—and determining whether deglycosylation is necessary. Tahir et al. formulated four OspA mRNA vaccine candidates delivered by lipid nanoparticles: native OspA (mRNA-OspA), OspA with a viral hemagglutinin secretion signal, deglycosylated OspA with a viral hemagglutinin secretion signal, and transmembrane OspA. *In vitro* tests revealed that antigen localization varied, occurring primarily intracellularly, in the supernatant, or transmembrane depending on the mRNA construct. Antigen localization influences the immune response elicited, and identifying the optimal candidate often involves trial and error.

To assess the efficacy of *B. burgdorferi* vaccines, two primary mouse challenge models are used to evaluate protection against bacterial infection: (1) subcutaneous injection of *B. burgdorferi* and (2) a tick-fed model using *B. burgdorferi*-infected ticks. Although the former injection model provides an excellent screening tool for vaccine efficacy, the tick-fed model is more efficient in spirochete transmission than injection, more accurately recapitulates the natural infection cycle, takes into consideration the role of bioactive saliva molecules that enhance transmission, and provides a more stringent challenge model.^4^ While the tick-fed mouse model has been extensively reported in the evaluation of protein subunit vaccines, the use of this model by Tahir et al. to rigorously evaluate mRNA-based vaccines helps support evidence that this vaccine approach may succeed beyond pre-clinical models.

*B. burgdorferi* is an extracellular pathogen, and humoral responses have been demonstrated to play a key role in borreliacidal effects and modulating infection, including spirochete blocking within the tick gut by antibody transfer during feeding. The mRNA-OspA vaccine elicited superior total OspA-specific immunoglobulin G (IgG) levels at 45 days post-vaccination compared to those with adjuvanted subunit vaccines, particularly enhancing IgG2a subclass titers, associated with a CD4 Th1 profile. Similar to subunit formulations, a decrease in OspA-specific titers was observed by day 70.

**Figure 1:**
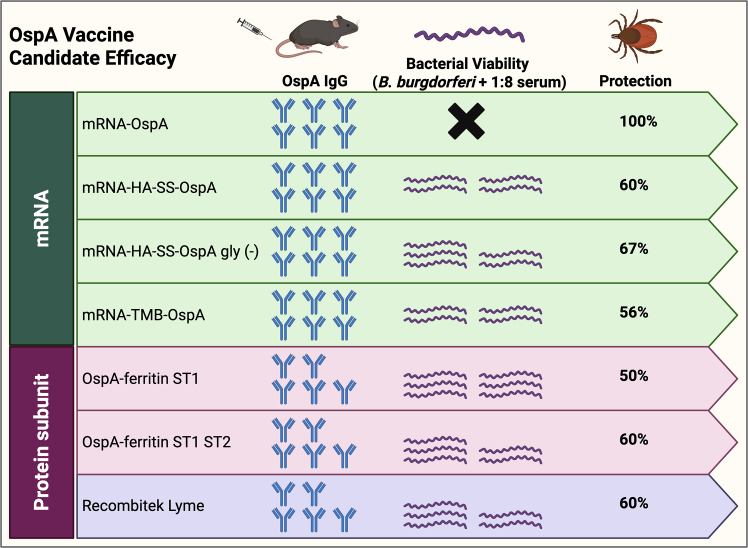
Candidate OspA mRNA and subunit vaccines and observed responses in a tick-fed mouse challenge model A summary of the humoral response, serum bactericidal assessment, and level of protection from infection post-challenge with *Borrelia burgdorferi* sensu stricto strain B31 in a C3H/HeNRj mouse model. The relative presence of outer surface protein A (OspA) IgG antibodies induced by vaccination is indicated by an antibody symbol; each symbol represents an approximately 1 log titer increase. The relative bacterial viability in a serum bactericidal assay is indicated by a spirochete symbol. Each spirochete symbol represents an approximately 1 log increase in viability, and the × symbol indicates no viability detected. Created with BioRender.com.

Despite high antibody titers observed in response to all mRNA vaccines, significant differences were found in the functionality of the antibodies generated from each mRNA vaccine construct. SARS-CoV-2 mRNA vaccines have been shown to require a signal peptide but not a transmembrane domain for a neutralizing antibody response to occur.^5^ Interestingly, the native mRNA-OspA vaccine expressing intracellular OspA without a signal peptide or transmembrane domain induced the highest bactericidal activity and was the only vaccine to provide complete (100%) protection (Figure 1). This vaccine significantly outperformed all other mRNA- and protein-based vaccines in both bactericidal antibody titers and protection from challenge. T cell responses were not evaluated in this study, though previous studies have shown that they are a crucial factor in protection as observed by an OspA-expressing DNA vaccine.^6^ T cell recognition of OspA has been shown to be a critical factor in generating protective antibody responses.^7^^,^^8^ It may be the case that superior protection is achieved by the mRNA-OspA vaccine through expression of an intracellular antigen that supports enhanced antigen processing by both the major histocompatibility complex class I and II pathways to achieve broad T cell responses compared to the other mRNA constructs.

The comparison with subunit OspA-Ferritin and Recombitek Lyme vaccines provided an important bridge to previously reported studies. While previous tick-fed challenge studies with subunit vaccines demonstrated 100% protection,^9^^,^^10^ under the specific challenge conditions used in this study (*Ixodes ricinus* tick species, exposure to 12 infected nymphs, and *B. burgdorferi* sensu stricto strain B31), around 60% of animals were protected against infection with subunit vaccines, compared to 100% with the mRNA-OspA vaccine. These findings underscore the importance of evaluating pre-clinical efficacy across multiple tick species and *Borrelia* stains/serotypes to identify the breadth of protection.

This study and the previous mRNA vaccine study against Lyme disease performed by Pine et al.^11^ demonstrated that mRNA vaccines can be highly effective and superior to subunit protein vaccines. However, the direct comparison between the two mRNA vaccines is challenging because the two studies used different immunization regimes, challenge models, and protection analyses.

Further research is needed to assess the long-term effectiveness of the mRNA-OspA vaccine against Lyme disease, as well as its cross-protective efficacy against other challenge strains. Additionally, recent advances in bivalent and multivalent mRNA vaccines, which generate strong humoral and cellular immune responses, underscore the potential advantages of incorporating multiple *Borrelia* antigens to enhance immunogenicity.

In summary, the study by Tahir et al. demonstrates the promising potential of the mRNA-OspA vaccine against Lyme disease. This study has confirmed that mRNA vaccines can be protective against bacterial infections; however, the antigen presentation requirements may be different from that typically used for viral antigens. This is an area of research worth further investigation. Overall, the native mRNA-OspA vaccine provided complete protection against *B. burgdorferi* strain B31 infection in a tick-fed mouse model, outperforming other tested mRNA and subunit vaccine candidates. As mRNA therapeutics continue to advance, it will be exciting to witness progression toward an approved Lyme disease vaccine in the future.

## Author contributions

W.H., A.R., N.O.F., J.Z.K.-S., and Z.R.S. wrote the commentary.

## Declaration of interests

The authors declare no competing interests.
